# Rare Presentation of Disseminated Histoplasmosis in an Immunocompetent Host

**DOI:** 10.4269/ajtmh.15-0239

**Published:** 2015-12-09

**Authors:** Anu Anna George, Mandeep Bindra, Promila Mohanraj

**Affiliations:** Christian Medical College, Vellore, Tamil Nadu, India

**Figure 1.**
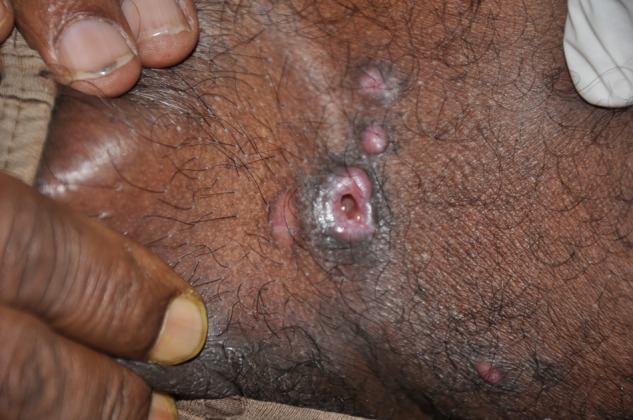
Ulcerated plaque in the groin of a patient with disseminated histoplasmosis.

A 40-year-old gentleman from West Bengal, who is a farmer by profession, presented with easy fatigability, significant loss of weight, and asymptomatic, progressive nodulo-ulcerative lesions on his groins of 1 year duration. He gave no history of exploring caves or exposure to any large areas contaminated with bird droppings. General examination revealed firm, non-tender, discrete cervical, axillary, and inguinal lymphadenopathy. Systemic examination was within normal limits. Cutaneous examination showed dull erythematous, non-tender, and firm papules and nodules, few with central ulceration on both groins (Figures 1

and 2

**Figure 2.**
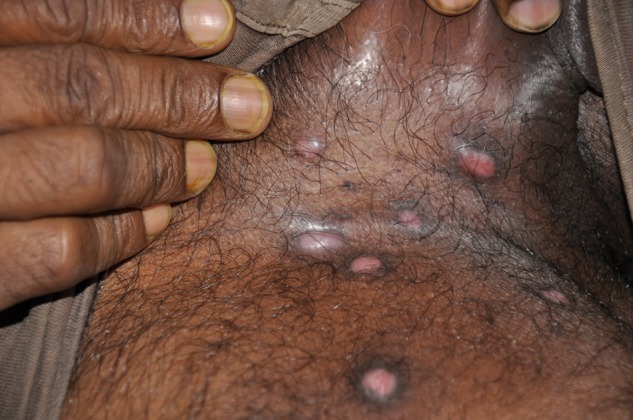
Erythematous, nodular lesions in the groin of a patient with disseminated histoplasmosis.

). There were no other skin lesions seen. The differentials considered were cutaneous tuberculosis, gummatous syphilis, atypical mycobacterial infection, nocardiosis, blastomycosis, sporotrichosis, and histiocytosis. A skin biopsy showed dense lymphoplasmahistiocytic infiltrates in the dermis with multinucleate giant cells containing budding yeasts (Figures 3

**Figure 3.**
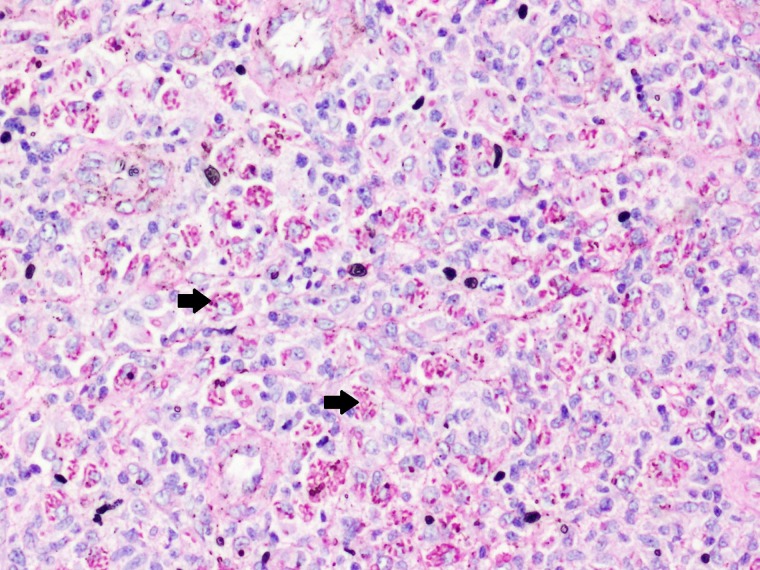
Periodic acid–Schiff (PAS) histopathology. Photomicrograph showing PAS-positive yeasts in the deep dermis.

and 4

**Figure 4.**
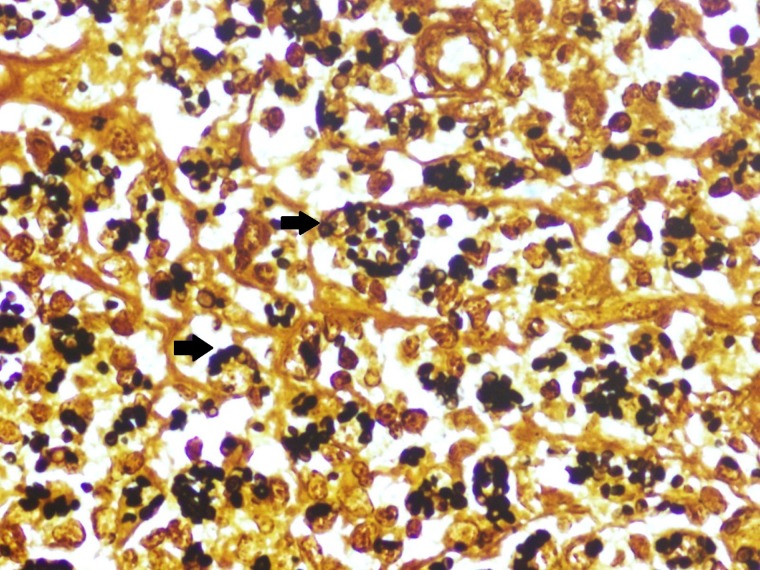
Gomori methanamine silver stain histopathology. Photomicrograph showing Gomori methanamine silver-positive yeasts in the deep dermis.

). Fungal culture on Sabouraud Dextrose Agar medium showed growth of floccose, velvety, buff colored colonies on the obverse with brown pigmentation on the reverse, which were confirmed to be *Histoplasma capsulatum* (Figures 5–7). Blood investigations to look for immunosuppression including human immunodeficiency virus antibodies were negative. A computerized tomography (CT) scan of the abdomen showed large hypodense lesions in both adrenals. His chest X-ray and bone marrow examination were within normal limits. A diagnosis of disseminated histoplasmosis was made based on the skin biopsy showing intracellular yeasts, which on culture grew colonies of *H*. *capsulatum*, and the CT scan showing involvement of both adrenals.

**Figure 5.**
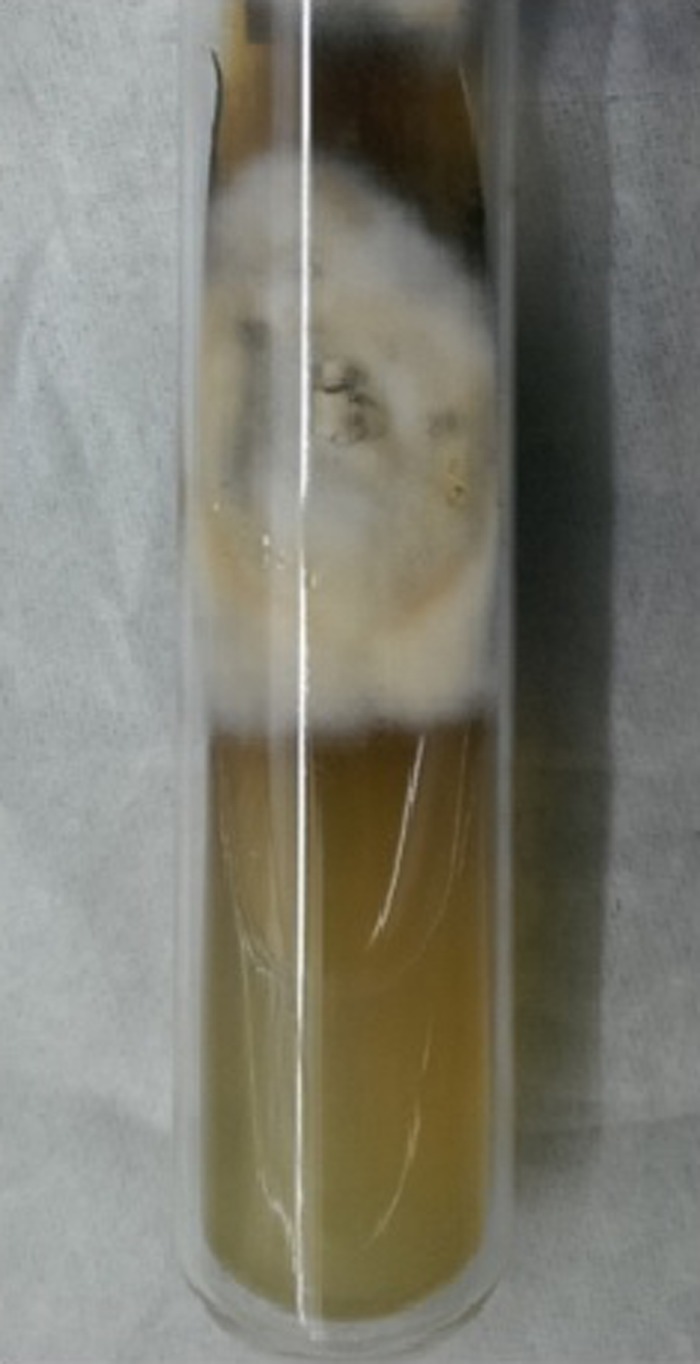
Sabouraud Dextrose Agar medium showing floccose, velvety, and buff colored colonies of *Histoplasma capsulatum* on the obverse.

**Figure 6.**
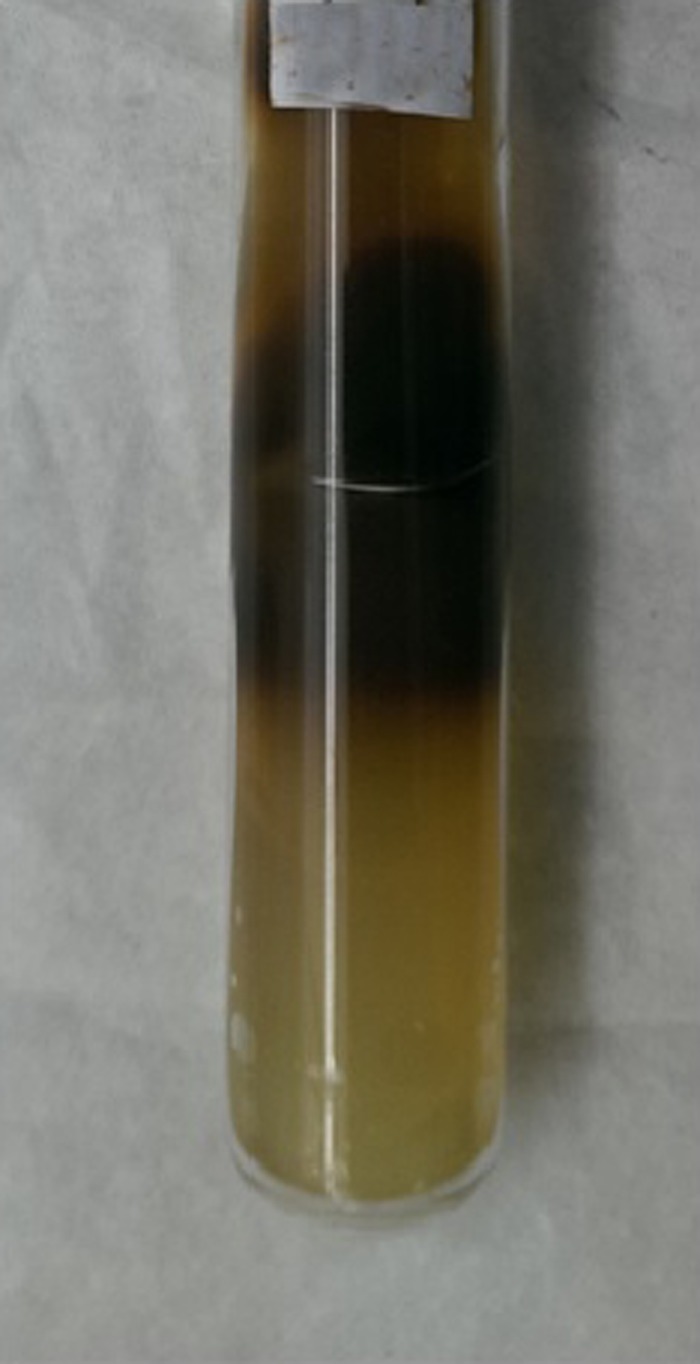
Sabouraud Dextrose Agar medium showing brown color on the reverse.

**Figure 7.**
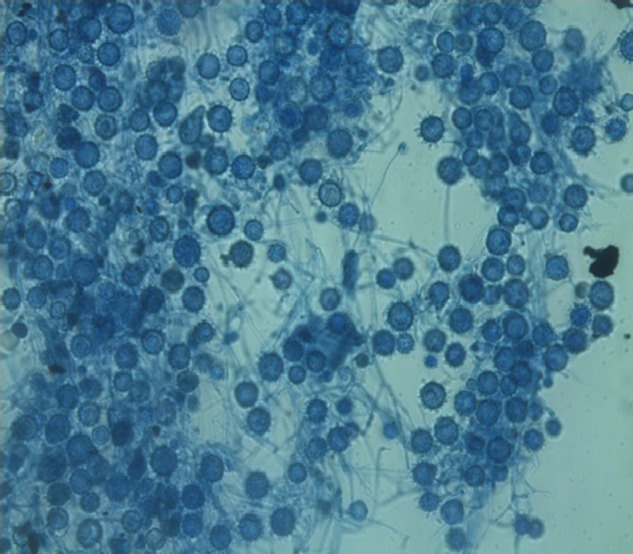
Lactophenol cotton blue preparation from the culture plate showing septate, hyaline hyphae with large thick-walled spherical macroconidia with the external walls showing finger-like projections.

Histoplasmosis is a highly infectious mycosis caused by an intracellular fungus *H*. *capsulatum*. Histoplasmosis is endemic in the Ohio and Mississippi River Valleys of the United States, central and South America, and Africa, but is less frequently reported in Asia and Europe.^1^ As pointed by Antinori,^2^ in India, this disease is predominantly seen along the Gangetic belt of northeast India. It can present as acute pulmonary, chronic pulmonary, acute progressive, chronic disseminated, and primary cutaneous forms. The risk factors for the acquisition of the disease include acquired immunodeficiency syndrome, primary and acquired immunodeficiency states, post transplant patients, and patients on immunosuppressive medications and extremes of age.^3^ In immunocompetent individuals, the disease usually manifests as an asymptomatic pulmonary infection. Disseminated histoplasmosis with cutaneous lesions is very rare in immunocompetent hosts.^1^ Oral ulcers are the most common mucocutaneous presentation in immunocompetent individuals with disseminated histoplasmosis.^4^ Cutaneous lesions of disseminated histoplasmosis are most commonly seen on the face, arms, and legs. The rarer sites of involvement, which have been reported include hands, feet, chest, back, penis, and perianal region.^5^ The oral lesions of histoplasmosis described in literature range from nodules, vegetating plaques to shallow or deep ulcers. The various cutaneous manifestations mentioned in literature include papules (including molluscum contagiosum–like lesions, acneiform eruptions), plaques, pustules, and nodules.^5^ This case is being reported to highlight the presentation of disseminated histoplasmosis at a rare site in an immunocompetent individual.
